# A cost-effectiveness analysis of the 13-valent pneumococcal conjugated vaccine and the 23-valent pneumococcal polysaccharide vaccine among Thai older adult

**DOI:** 10.3389/fpubh.2023.1071117

**Published:** 2023-06-29

**Authors:** Thundon Ngamprasertchai, Chayanis Kositamongkol, Saranath Lawpoolsri, Pinyo Rattanaumpawan, Viravarn Luvira, Piriyaporn Chongtrakool, Jaranit Kaewkungwal, Kulkanya Chokephaibulkit, Pochamana Phisalprapa

**Affiliations:** ^1^Department of Clinical Tropical Medicine, Faculty of Tropical Medicine, Mahidol University, Bangkok, Thailand; ^2^Division of Ambulatory Medicine, Department of Medicine, Faculty of Medicine Siriraj Hospital, Mahidol University, Bangkok, Thailand; ^3^Department of Tropical Hygiene, Faculty of Tropical Medicine, Mahidol University, Bangkok, Thailand; ^4^Department of Medicine, Faculty of Medicine Siriraj Hospital, Mahidol University, Bangkok, Thailand; ^5^Department of Microbiology, Faculty of Medicine Siriraj Hospital, Mahidol University, Bangkok, Thailand; ^6^Department of Pediatrics, Faculty of Medicine Siriraj Hospital, Mahidol University, Bangkok, Thailand; ^7^Siriraj Institute of Clinical Research, Faculty of Medicine Siriraj Hospital, Mahidol University, Bangkok, Thailand

**Keywords:** 13-valent pneumococcal conjugated vaccine (PCV13), 23-valent pneumococcal polysaccharide vaccine (PPSV23), cost-effectiveness, economic evaluation, Thailand, older adult

## Abstract

**Introduction:**

This study aims to assess the economic impact of introducing the 13-valent pneumococcal conjugate vaccine (PCV13) and 23-valent pneumococcal polysaccharide vaccine (PPSV23) to Thai older adult aged ≥ 65  years who are healthy or with chronic health conditions and immunocompromised conditions from a societal perspective in order to introduce the vaccine to Thailand’s National Immunization Program for the older adult.

**Methods:**

A Markov model was adopted to simulate the natural history and economic outcomes of invasive pneumococcal diseases using updated published sources and Thai databases. We reported analyses as incremental cost-effectiveness ratios (ICER) in USD per quality-adjusted life year (QALY) gained. In addition, sensitivity analyses and budget impact analyses were conducted.

**Results:**

The base-case analysis of all interventions (no vaccinations [current standard of care in Thailand], PPSV23, and PCV13) showed that PPSV23 was extendedly dominated by PCV13. Among healthy individuals or those with chronic health conditions, ICER for PCV13 was 233.63 USD/QALY; meanwhile, among individuals with immunocompromised conditions, ICER for PCV13 was 627.24 USD/QALY. PCV13 are economical vaccine for all older adult Thai individuals when compared to all interventions.

**Conclusions:**

In the context of Thailand, PCV13 is recommended as the best buy and should be primarily prioritized when both costs and benefits are considered. Also, this model will be beneficial to the two-next generation pneumococcal vaccines implementation in Thailand.

## 1. Introduction

Two next-generation higher valency pneumococcal conjugate vaccines (15-valent or 20-valent pneumococcal conjugate vaccines, PCV15 or PCV20) are expected to be implemented for adult use in high-income countries in the near future before pediatric licensing (1). However, Thailand has not yet to incorporate any pneumococcal vaccines into the National Immunization Program (NIP) as of 2022 (2). There is strong evidence that pneumococcal vaccines reduce the incidence of invasive pneumococcal diseases (IPDs) or pneumococcal diseases (3). IPDs afflicted not only young children but also the older adult. In Thailand, the incidence of IPDs among older adult was 26 per 100,000 individuals (4), and the hospitalization rate was 30.5 per 100,000 person-years (5). Total costs by age groups for all types of pneumococcal diseases ranged from 680.7 million USD to 1,346.7 million USD in 2009 with the highest cost among patients aged 75–84 years (6). While, Thai children would lose 453,401 years of life and 457,598 productivity-adjusted life years (PALYs) which were equivalent to 5,586 USD million to pneumococcal diseases (7). Introducing a pneumococcal vaccine is an effective strategy for reducing disease-burden nasopharyngeal carriage and generating an indirect herd effect in the community.

In recent years, there has been much discussion about including pneumococcal vaccines in Thai NIP. This is because policymakers must weigh the costs and benefits of vaccination. The major considerations before vaccine implementation are that current economic evaluations are limited to pediatric populations and yield conflicting results. Although studies among children from the Philippines and Singapore, demonstrated that implementing pneumococcal vaccine in the health system was cost-effective compared with no vaccination program (8, 9), several studies from Thailand focused on children and yielded conflicting results. Kulpeng et al. (10) demonstrated that in 2013, the 10-valent pneumococcal polysaccharide nontypeable *Haemophilus influenzae* protein D-conjugated vaccine (PHiD-CV) and PCV13 were not cost-effective when compared with no vaccine due to their high costs. In 2019, Dilokthornsakul et al. (11) incorporated herd immunity effects and showed that both vaccines were considered cost-effective among Thai children. However, economic studies focusing on Thai older adult are scarce.

According to some economic studies, either PCV13 or the 23-valent pneumococcal polysaccharide vaccine (PPSV23) is cost-effective among the older adult. Igarashi et al. (12) performed a cost-effectiveness analysis of PCV13 and PPSV23 in older adult aged ≥ 60 years in a Japanese health care setting. The study showed that PCV13 was more cost-effective than PPSV23 (12). Meanwhile, the study conducted in Denmark suggested that implementing a PPSV23 vaccination program for all older adult aged ≥ 65 years is cost-effective (13). Even though there are many economic studies on the older adult from various countries, the generalizability of the results should be considered due to each country’s diverse backgrounds such as pneumococcal serotype distributions and vaccine support from non-profit organization.

Adult pneumococcal vaccine policies vary across countries as a result of epidemiological changes in the disease burden caused by the indirect effect of child vaccination programs. Currently, the Thai older adult have two options for pneumococcal vaccine: (1) PCV13 and (2) PPSV23. The (14) suggests older adult immunization schedules, either PCV13 or PPSV23, among older adult aged ≥ 65 years with or without chronic health conditions or immunocompromised conditions. Meanwhile, the Pediatric Infectious Disease Society of Thailand (15) recommend either PCV10 or PCV13 as an optional vaccine for healthy children aged 2, 4, and 12–15 months. Even though two pneumococcal vaccines are available in Thailand, disease burden remains high while vaccine uptake rate is low (16). All Thai individuals need to afford the out-of-pocket costs of pneumococcal vaccines regardless of their immune status as the vaccines are not listed in NIP. The cost of vaccines is the significant barrier to national implementation in Thailand; therefore, negotiations with manufacturers is encouraged. Using available local data, this study aimed to perform an economic evaluation of both PCV13 and PPSV23 compared with no vaccine and each other among Thai older adult aged ≥ 65 years in order to introduce the vaccine to Thailand’s NIP for the older adult.

## 2. Methods

We conducted a cost-effectiveness analysis to determine whether the benefits derived from the different vaccine interventions [no vaccinations (current standard of care in Thailand), PPSV23, and PCV13], measured as quality-adjusted life year (QALY) gained, offered value for money. We applied a decision rule for cost-effectiveness analysis as mentioned in Karlsson et al. (17) to compare the cost-effectiveness of interventions and excluded dominated vaccines from the analyses. If a vaccine was less effective and more costly than other interventions or the incremental cost-effectiveness ratio (ICER) was higher when compared to the next more effective vaccine will be dominated (known as extended dominance). This study defined the older adult population as those aged 65 years and older. Currently, Thailand’s NIP does not include pneumococcal vaccination for any age group. We classified targeted populations into two groups based on pneumococcal vaccine recommendations (18): (1) healthy individuals and/or those with chronic health conditions (i.e., chronic heart, lung, or liver diseases) and (2) individuals with immunocompromised conditions (i.e., HIV infection, lymphoma, or generalized malignancy). The information regarding chronic and immunocompromised conditions was defined by Matanock et al. (18). The lifetime horizon was selected for the analysis because some cases of IPDs have long-term effects, such as hearing loss or neurofunctional impairment after meningitis. The annual discount rate of 3% was applied to both costs and outcomes. We presented an ICER in United State dollars (USD) per QALY gained from the perspective of Thai society.

### 2.1. Model overview

Based on disease characteristics, vaccine effectiveness and previous literature (19, 20), this study developed a Markov model. The model’s cycle length was set to 1 year. The virtual cohort was followed on an annual basis until death. We assumed that all individuals who were not infected would have the same life expectancy as the general Thai population and all transition states were mutually exclusive (19). Once he or she had pneumococcal infection, the mortality rate would change depending on the condition. Our model took into account four conditions/consequences following a pneumococcal infection: (1) meningitis, (2) bacteremia without pneumonia, (3) bacteremia with pneumonia, and (4) pneumococcal pneumonia. Disseminated infection and adverse events associated with PCV13 and PPSV23 vaccination were not considered because they were of rare condition and mild or moderate severity, respectively (21, 22).

**Figure 1:**
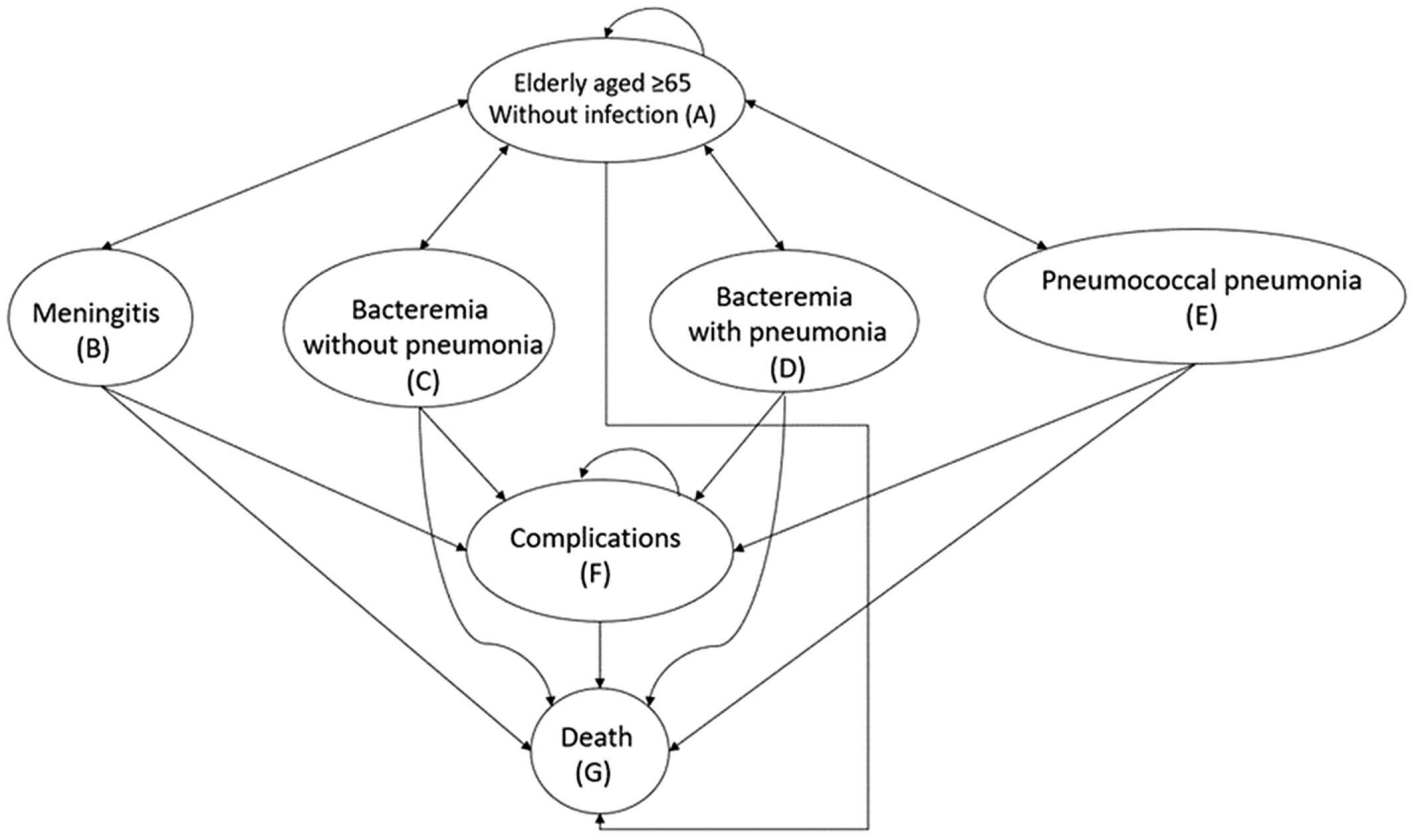
Markov model for pneumococcal vaccination and infection. Health states are in oval shapes. Transitions between states or remaining in the same state are represented by arrows.

Our Markov model has seven health states that are mutually exclusive (Figure 1). All virtual patients would enter the model in the non-infection state (State A: older adult aged ≥ 65 years without infection). They could then either (1) become infected with pneumococcal bacteria and progress to one of the four health states (States B–E) that represent the conditions/results of pneumococcal bacterial infection, (2) remain in the same non-infection health state (State A), or (3) die (State G). The model allowed for some patients to experience complications following the infection (State F), whereas others would fully recover and return to the non-infection state. The complications were permanent conditions such as hearing loss, neurofunctional impairment following meningitis, and chronic lung diseases after pneumonia. The arrows in Figure 1 depict transitions between health states and staying in the same states. Transitions from a non-infection health state to a pneumococcal disease state were determined by the subjects’ health status, the incidence of each consequence and the effect of vaccination.

### 2.2. Model input parameters

#### 2.2.1. Epidemiology data

The input variables were obtained from published literature (Table 1). Meningitis, bacteremia without pneumonia, and bacteremia with pneumonia incidence rates were calculated separately based on the incidence of IPDs among Thai individuals with and without chronic health conditions. The rates were derived from a population-based survey in Sa Kaeo and Nakhon Phanom provinces (located in Northeast of Thailand) (4). Incidence of IPDs among individuals with immunocompromised conditions was derived from Smith et al. (20) using data collected in the United States prior to the 2010 introduction of the pneumococcal vaccine. In our model, we assumed that three IPD consequences were mutually exclusive and that the probabilities of transitioning from a non-infected state to meningitis, bacteremia without pneumonia, and pneumococcal bacteremia were proportional. The ratio was derived from previously published articles (20) that report the age-specific value of individuals stratified by their medical conditions. Case fatality rate were derived using data from Asia-Pacific countries (24) and assumed with an odds ratio for immunocompromised conditions between 1.3 and 1.8. This similar approach was used in the previous published economic analysis (12, 20). The proportion of IPD outcomes and incidence of pneumococcal disease complications were assumed to be equal for infected healthy individuals, those with chronic health conditions and those with immunocompromised conditions. We extracted the data from Smith et al. (20) and Hoshi et al. (19) as there was evidence that incidence rates of pneumococcal pneumonia without bacteremia and its complications (chronic lung diseases following infection) were higher in immunocompromised conditions than in healthy older adult. The incidence of adverse events was excluded for both PCV13 and PPSV23 because the majority was minimal, non-serious and indifferent from placebo (25, 34, 35).

**Table 1:** Input parameters.

Parameters	Healthy or chronic health conditions		Immunocompromised conditions		Distribution	References
Epidemiology/efficacy/effectiveness parameters	Mean	± Range	Mean	± Range		
Incidence of IPDs (per 100,000 population per year)
Incidence of meningitis	2.48	0.12	9.1	0.45	normal	(20)
Incidence of bacteremia	13.6	0.68	17.39	0.87	normal	(23)
Incidence of bacteremia with pneumonia	9.92	0.50	22.37	1.12	normal	(20)
Fatality rate fatality rate of IPDs (%)	29.1 (3.9–54.3)	1.46	43.65		normal	(24)
Case fatality odds ratio for immunocompromised conditions			1.5	0.3	Log normal	(12, 20)
**IPD complications** Epilepsy after meningitis	0.082	0.004	0.082	0.004	normal	(11)
Hearing loss after meningitis	0.016	0.0008	0.016	0.0008	normal	
Neurofunctional impairment after meningitis	0.002	0.00009	0.002	0.00009	normal	
Complication from bacteremia	0.070	0.004	0.070	0.004	normal	(19)
Complication from bacteremia with pneumonia	0.020	0.001	0.020	0.001	normal	
Death after complication	0.050	0.003	0.050	0.003	normal	
**Non-bacteremic pneumococcal** pneumonia incidence (per 100,000 population per year)	284.9	14.25	871.45	43.57	normal	(20)
Complications	0.027	0.001	0.027	0.001	normal	(19)
Fatality rate of pneumococcal pneumonia (%)	23.15 (1.9–44.4)	1.16	34.73	-	normal	(24)
Case fatality odds ratio for immunocompromised conditions			1.5	0.3	Log normal	(12, 20)
Vaccine efficacy protection against IPDs caused by vaccine serotype (PCV13) Duration of protection: no decline for 5 years then decline to 0 over 10 years (25)						
0	0.750	0.171	0.250	0.057	normal	(25) (Healthy or chronic health conditions) For immunocompromised conditions: Assumed 1/3 of healthy/chronic medical conditions adults (26)
6	0.675	0.154	0.225	0.051	normal	
7	0.600	0.137	0.200	0.046	normal	
8	0.525	0.120	0.175	0.040	normal	
9	0.450	0.103	0.150	0.034	normal	
10	0.375	0.086	0.125	0.029	normal	
11	0.300	0.069	0.100	0.023	normal	
12	0.225	0.051	0.075	0.017	normal	
13	0.150	0.034	0.050	0.011	normal	
14	0.075	0.017	0.025	0.006	normal	
15	0.000	0.000	0.000	0.000	normal	
Protection against pneumonia caused by vaccine serotype (PCV13)						
0	0.535	0.214	0.150	0.053	normal	(27) (Healthy or chronic health conditions) For immunocompromised conditions: Assumed 1/3 of healthy/chronic medical conditions adults (26)
6	0.482	0.192	0.135	0.047	normal	
7	0.428	0.171	0.120	0.042	normal	
8	0.375	0.150	0.105	0.037	normal	
9	0.321	0.128	0.090	0.032	normal	
10	0.268	0.107	0.075	0.026	normal	
11	0.214	0.086	0.060	0.021	normal	
12	0.161	0.064	0.045	0.016	normal	
13	0.107	0.043	0.030	0.011	normal	
14	0.054	0.021	0.015	0.005	normal	
15	0.000	0.000	0.000	0.000	normal	
Vaccine efficacy protection against IPDs caused by vaccine serotype (PPSV23) Duration of protection: Linear decline for 0 over 15 years						
0	0.597	0.063	0.079	0.134	normal	Meta-analysis (28) (29) (30)
1	0.557	0.059	0.074	0.125	normal	
2	0.517	0.054	0.068	0.116	normal	
3	0.478	0.050	0.063	0.107	normal	
4	0.438	0.046	0.058	0.098	normal	
5	0.398	0.042	0.053	0.089	normal	
6	0.358	0.038	0.047	0.081	normal	
7	0.318	0.033	0.042	0.072	normal	
8	0.279	0.029	0.037	0.063	normal	
9	0.239	0.025	0.032	0.054	normal	
10	0.199	0.021	0.026	0.045	normal	
11	0.159	0.017	0.021	0.036	normal	
12	0.119	0.013	0.016	0.027	normal	
13	0.080	0.008	0.011	0.018	normal	
14	0.040	0.004	0.005	0.009	normal	
15	0.000	0.000	0.000	0.000	normal	
Protection against pneumonia caused by vaccine serotype (PPSV23)						
0	0.200	0.102	0.067	0.034	normal	(31) (Healthy or chronic health conditions) For immunocompromised conditions: Assumed 1/3 of healthy/chronic medical conditions adults (26)
1	0.187	0.095	0.063	0.032	normal	
2	0.173	0.088	0.058	0.030	normal	
3	0.160	0.082	0.054	0.027	normal	
4	0.147	0.075	0.049	0.025	normal	
5	0.133	0.068	0.045	0.023	normal	
6	0.120	0.061	0.040	0.021	normal	
7	0.107	0.054	0.036	0.018	normal	
8	0.093	0.048	0.031	0.016	normal	
9	0.080	0.041	0.027	0.014	normal	
10	0.067	0.034	0.022	0.011	normal	
11	0.053	0.027	0.018	0.009	normal	
12	0.040	0.020	0.013	0.007	normal	
13	0.027	0.014	0.009	0.005	normal	
14	0.013	0.007	0.004	0.002	normal	
15	0.000	0.000	0.000	0.000	normal	
Utility parameters						
Utility for Thai older adult	0.834	0.003	0.626	0.002	Beta	(32) (19) (33)
Utility for meningitis	0.400	0.040	0.400	0.040	Beta	
Utility for bacteremia	0.500	0.050	0.500	0.050	Beta	
Utility for pneumonia	0.500	0.050	0.500	0.050	Beta	
Utility for neurological and chronic lung complications	0.300	0.030	0.300	0.030	Beta	
Cost parameters Vaccine cost PCV13 (USD/vial)	64.71				Fixed	(38)
PPSV23 (USD/vial)	29.44				Fixed	
Administration cost PCV13	3.23				Fixed	Assumption
PPSV23	3.23				Fixed	
Direct medical costs Cost per episode Meningitis	3,490.84	199.47	3490.84	199.47	Gamma	(11)
Bacteremia	2419.04	121.14	2419.04	121.14	Gamma	
Pneumonia	2881.49	272.55	2881.49	272.55	Gamma	
Cost per year						
Epilepsy	451.44	4.03	451.44	4.03	Gamma	
Hearing loss	27.70	0.31	27.70	0.31	Gamma	
Neurological impairment	41.46	2.44	41.46	2.44	Gamma	
Chronic lung	122.98	1.00	122.98	1.00	Gamma	

IPD: Invasive Pneumococcal Vaccine; PCV: Pneumococcal Conjugate Vaccine; PPS: Pneumococcal Polysaccharide Vaccine.

#### 2.2.2. Vaccine efficacy/effectiveness

We adopted the vaccine effectiveness model based on the Centers for Disease Control and Prevention (CDC) model, which was updated in the Advisory Committee on Immunization Practices meeting 2021 (36). According to various resources, the CDC model included both clinical trial phases 3 and 4. Therefore, either vaccine efficacy or effectiveness was present in the input parameters. The duration of PCV13 protection is 15 years, with no decline for the first 5 years, but declines linearly to 0 after 10 years. However, the protection duration of PPSV23 has been decreasing linearly since the first year, and its protective effect will be zero in 15 years. Because pneumococcal vaccine has not yet been implemented in Thailand’s NIP, the indirect effect of vaccine was not considered in our model.

#### 2.2.3. Utilities

The utility of Thai healthy individuals and those with chronic health conditions in the absence of infection was based on the average utility of Thai adults aged 45–70 years. We estimated the utility for immunocompromised older adult without infection proportionally based on the utility ratio of populations at high and average risk in the same age range (20). In the absence of local data regarding the utility of adults with infection, we based our model on the National Health Interview Survey data from 1990 to 2000 from the resident civilian non-institutionalized population of the United States. The utility for episode of infection was assumed to be equivalent in both scenarios.

#### 2.2.4. Costs

Because the model was developed from a societal standpoint, direct medical and non-medical costs were included. We exclude indirect costs because we assumed that lost or impaired ability to work or engage in leisure activities due to morbidity would be accounted for in the disutility of QALY (37). PCV13 and PPSV23 prices were obtained from the Drug and Medical Supply Information Center (38). The cost of vaccination acquisition and wastage was obtained from a Thailand’s survey study (39) (in Thai). The cost of vaccine administration was 3.23 USD which was referred to the standard cost list for health technology assessment and adjusted with consumer price index (40). We refer to a published cost-effectiveness study using the NHSO database (2011–2016) (11) to estimate age-specific treatment costs for bacteremia, meningitis, pneumonia and complications. We anticipated that all four infection conditions would necessitate hospitalization. Direct non-medical costs, such as transportation, meals, accommodation, and facilities, were derived from a previous published cost-effectiveness study (11). The cost per episode for healthy or chronic health conditions was assumed to be the same as the cost for immunocompromised conditions. The average of meningitis and pneumonia complications was used to estimate bacteremia complications. We applied consumer price index to convert the value of all past costs to the value of 2021. All costs were converted to USD using the official exchange rate of the world bank of Thai Baht (THB) 31.98 = 1 USD.

### 2.3. Base-case analysis

The incremental costs, life year gained, QALYs gained, and ICER were the study outcomes. For the base-case analysis, the expected lifetime costs and health-related outcomes of all interventions (no vaccinations, PPSV23, and PCV13) were presented and selected as comparators with the least lifetime costs and outcomes.

In our study, two scenarios were categorized by the target population of the vaccination program. The first scenario was based on healthy older adult with or without chronic health conditions. The latter study examined the cost-effectiveness of vaccines in immunocompromised individuals. Each scenario featured two pairs of comparisons. The willingness-to-pay (WTP) threshold of 5,003 USD/QALY gained according to the Thai Health Economic Working Group for drug listing in the National List of Essential Medicines in 2012 (41) determined the results of the cost-effectiveness analysis.

### 2.4. Sensitivity analyses

We ran sensitivity analyses to determine how ICER would change as a result of parameter uncertainty. In each scenario, one-way and probabilistic sensitivity analyses were performed. The lower and upper limits of the parameter values used in one-way sensitivity analyses were determined using each parameter’s standard error. Because the standard errors of incidence proportions and utilities were not available, we varied them within the range of ± 5 and ± 10% from the base-case values, respectively. The results of one-way sensitivity analyses were represented as tornado diagrams. The effect of all variable’s uncertainty was examined using probabilistic sensitivity analyses (PSA). We ran a Monte Carlo simulation in Microsoft Excel 2018 for 1,000 iterations to demonstrate a range of total cost, health-related outcomes, and ICER values. The PSA results were illustrated as cost-effectiveness planes and cost-effectiveness acceptability curves. Threshold analyses were also performed to determine the vaccines’ acceptable costs in Thailand. The analyses were carried out by lowering vaccine prices (USD per vial) until the ICER of each scenario became cost-effective (i.e., ≤ 5,003 USD/QALY gained) or cost-saving (i.e., 0 USD/QALY gained).

### 2.5. Budget impact analysis

We analyzed the 5-year budget impact to estimate the total budget for the national PCV13 and PPSV23 vaccination program for Thai older adult in 2022–2026. The total number of Thai older adult population aged ≥ 65 years were retrieved from the database of the Official Statistic Registration System, Department of Provincial Administration, Thailand (42). Prior to the implementation of NIP, approximately 15% of the older adult population in Thailand had been vaccinated, as alternative vaccines had been available in Thailand since 2011 (43). In Southeast Asia, however, the pneumococcal vaccine uptake rate was approximately 29%, which was lower than other regions (16). The acceptance rate of pneumococcal vaccines in the Thai population was estimated using the acceptance rate of influenza vaccine. According to published data, between 40 and 50% of the Thai population had received a flu vaccine (44). We predicted that the vaccines access rate would increase by 10% annually. We calculated the budgetary impact of two policy strategies: (1) ‘active policy strategy’ which permitted vaccination of the population aged ≥ 65 years (those who aged more than 65 years were supported by Thai government) and (2) ‘passive policy strategies,’ which allowed population to receive the vaccine only at the age of 65 years (only those at 65 years were subsidized by Thai government).

## 3. Results

### 3.1. Base-case analysis

**Figure 2:**
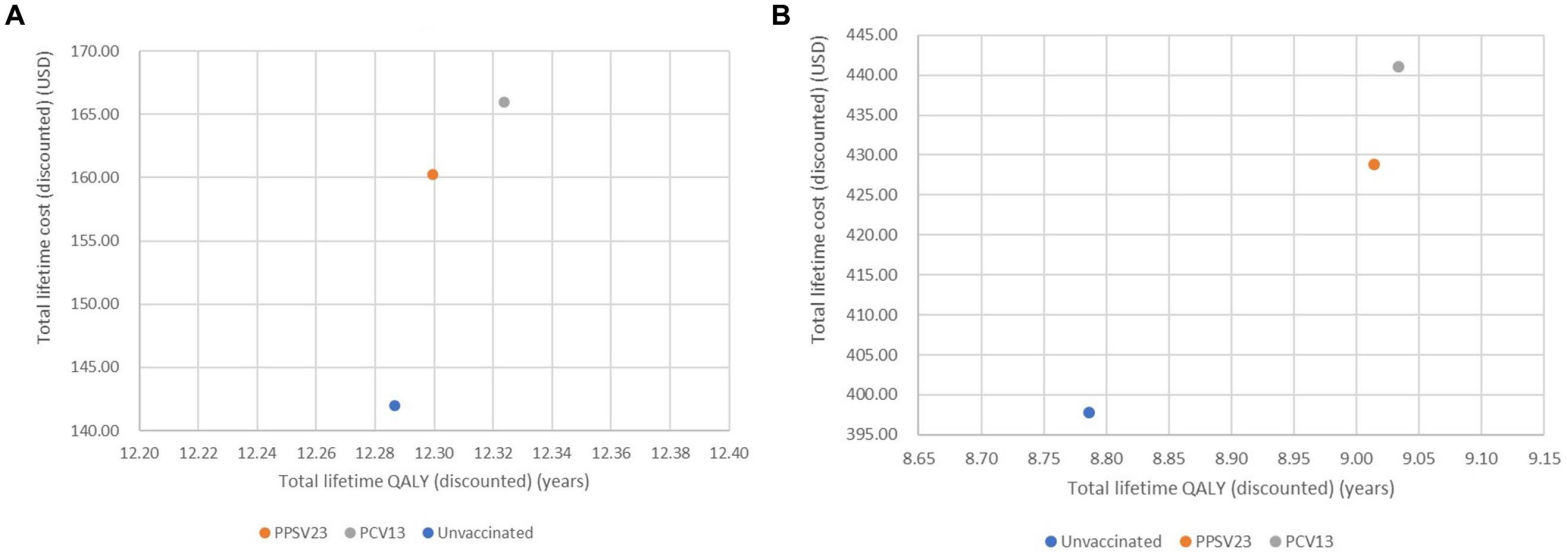
Cost-effectiveness plane of all interventions analysis of healthy or with chronic health conditions **(A)** older adult with immunocompromised conditions **(B)**.

In healthy or chronic health conditions, total lifetime QALYs of no vaccination, PPSV23, and PCV13 were 12.29, 12.30, and 12.32 years, respectively. Total lifetime costs of no vaccination, PPSV23, and PCV13 were 142.0, 160.3, and 166.0 USD, respectively. Meanwhile, total lifetime cost and QALY of PCV13 in immunocompromised conditions were greater than PPSV23 and then no vaccination (Figures 2A,B). Therefore, no vaccination was described as the comparator with the least total lifetime cost and QALYs, followed by PPSV23 and PCV13.

For one-time PPSV23 vaccination programs increased both cost and QALYs compared to no vaccination policy among healthy or chronic health conditions (Table 2). The incremental cost, life year, QALYs gained, and ICER of PPSV23 were 18.27 USD, 20.64 years, 0.01 QALYs, and 1439.25 USD/QALY gained, respectively. PPSV23 was extendedly dominated by PCV13 as PCV13 yielded more 0.02 QALYs/lifetime per individual with an incremental total lifetime cost of 5.67 USD/individual compared to PPSV23; hence the ICER of PCV13 vs. PPSV23 was 233.63 USD/QALY gained.

**Table 2:** Base-case analyses.

Vaccine	Total cost (USD)	LYs (years)	QALYs	Incremental cost (USD)	Incremental QALY	ICER
*Healthy or chronic health conditions*						
No vaccine	142.00	20.62	12.29			
PPSV23	160.27	20.64	12.30	18.27	0.01	1,439.25
PCV13	165.94	20.68	12.32	5.67	0.02	233.63
*Immunocompromised conditions*						
No vaccine	397.83	19.42	8.79			
PPSV23	428.81	20.10	9.01	30.98	0.23	136.13
PCV13	441.12	20.15	9.03	12.31	0.02	627.24

LYs: life years; QALYs: quality-adjusted life years; ICER: incremental cost-effectiveness ratio; PCV: pneumococcal conjugate vaccine; PPSV: pneumococcal polysaccharide vaccine.

In the scenario focusing on immunocompromised persons, the incremental cost, life year, QALYs gained, and ICER of PPSV23, compared with no vaccination, was 30.98 USD, 20.10 years, 0.23 QALYs and 136.13 USD/QALY gained, respectively. In addition, PCV13 would gain 0.02 QALYs/individual while costing 12.31 USD more per individual than PPSV23, resulting in an ICER of 627.24 USD/QALY gained. Comparing PCV13 with PPSV23 among healthy older adult, older adult with chronic health conditions and immunocompromised people, we found that PCV13 was a cost-effective vaccine, with an ICER of 233.63USD/QALY gained and 627.24 USD/QALY gained, respectively.

### 3.2. Sensitivity analyses

#### 3.2.1. One-way sensitivity analyses

**Figure 3:**
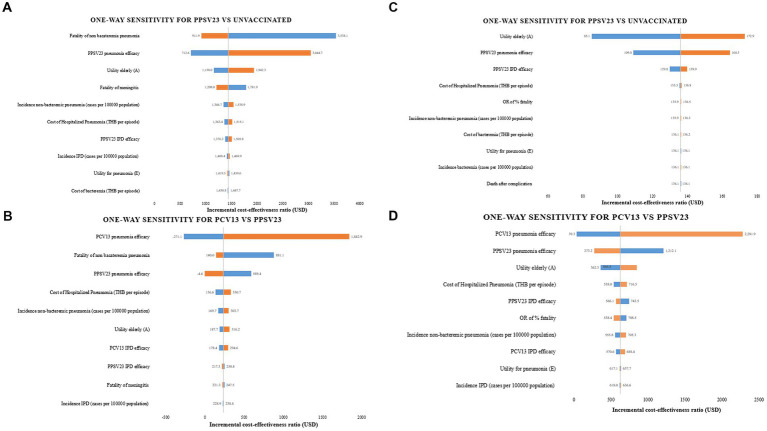
One-way sensitivity analyses. **(A)** PPSV23 vs. unvaccinated in healthy or chronic health conditions **(B)** PPSV23 vs. PCV13 in healthy or chronic health conditions **(C)** PPSV23 vs. unvaccinated in immunocompromised conditions **(D)** PPSV23 vs. PCV13 in immunocompromised conditions.

Case fatality of non bacteremic pneumococcal pneumonia and PCV13 efficacy protection against pneumonia were found to be the most sensitive input parameter in one-way sensitivity analyses of PPSV23 compared to no vaccination and PCV13, respectively (Figures 3A,B) that focused on older adult with healthy or chronic health conditions. The utility of the older adult in a non-infected health state was the input parameter that had the greatest impact on the results of immunocompromised older adult in PPSV23 vs. no vaccination. PCV13 efficacy protection against pneumonia demonstrated higher sensitivity parameters than PPSV23 when compared in all types of older adult (Figures 3C,D).

#### 3.2.2. Multivariate probabilistic sensitivity analyses

**Figure 4:**
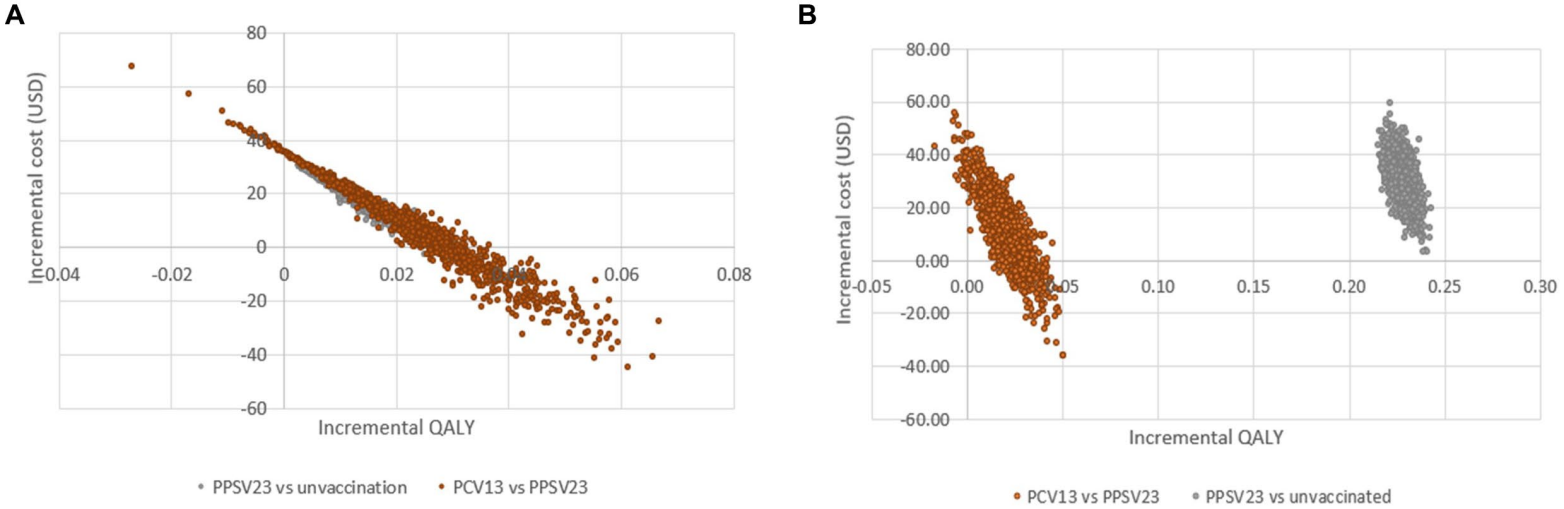
Cost-effectiveness scatter plot demonstrating probabilistic sensitivity analysis of healthy or with chronic health conditions **(A)** older adult with immunocompromised conditions **(B)**.

**Figure 5:**
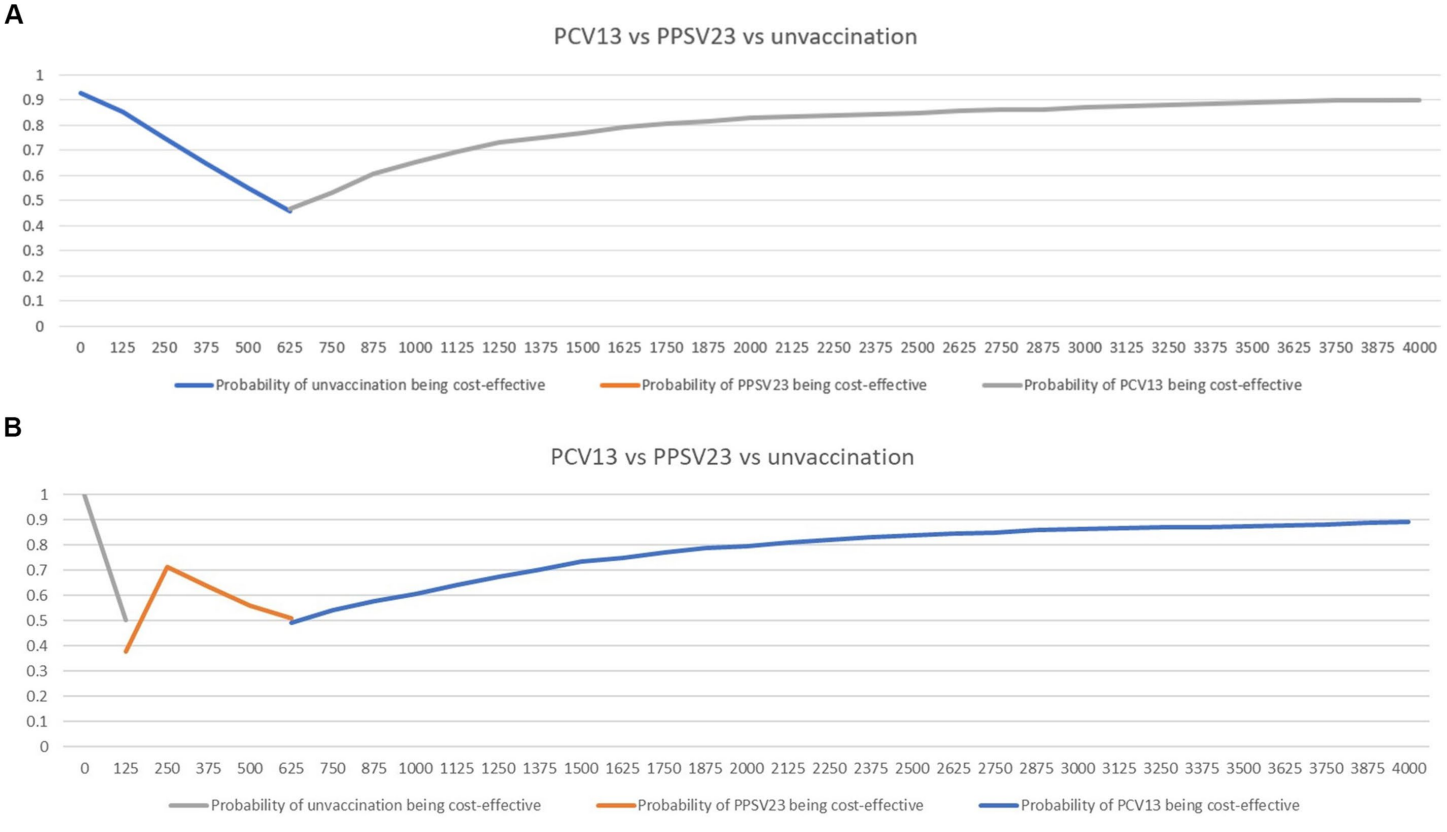
Cost-effectiveness acceptability curves showing the probability of being cost-effective and cost-saving when comparing PCV13 and PPSV23 among healthy or chronic health conditions **(A)** or immunocompromised conditions **(B)**.

The cost-effectiveness planes displayed PSA results based on 1,000 Monte Carlo simulations (Figure 4). In all scenarios, the majority of simulated ICERs were in the upper-right quadrant. These findings showed that PCV13 policy implementation provided higher QALYs at a higher cost than PPSV23 policy among all groups of Thai older adult. When compared to PPSV23, the probability of PCV13 being cost-saving among immunocompromised people was more than 90%, which was higher than the probability of healthy or chronic health conditions (80%; Figure 5).

#### 3.2.3. Threshold analysis

Thai Health Technology Assessment guidelines recommended that the result of disease prevention policies, such as vaccination program, should be cost-saving compared with no policy to consider adopting the policy for implementation. In terms of cost-saving, the cost of PCV13 vaccination needed to be reduced by 8.8 and 19.0% of the current price, when compared to PPSV23 among healthy older adult or older adult with chronic health conditions and people with immunocompromised conditions, respectively (Table 3).

**Table 3:** Threshold analyses.

Vaccination	PPSV23	PCV13	PPSV23 *vs* PCV13
Actual cost per vial (USD)	29.44	64.71	
*Healthy or chronic health conditions*			
% Reduction	62.10		8.80
Reduced cost per vial (USD)	11.17		59.04
*Immunocompromised conditions*			
% Reduction	N/A^*^		19.00
Reduced cost per vial (USD)	N/A^*^		52.40

* Not applicable unless the cost of vaccine administration was reduced.

PCV: pneumococcal conjugate vaccine; PPSV: pneumococcal polysaccharide vaccine; N/A: not applicable.

### 3.3. Budget impact analysis

Budget impact analysis (BIA) revealed that the Thai government may have to invest approximately 12–19 million USD per year for PCV13 implementation in passive policy strategies; however, the budget for PPSV23 policy implementation was lower than twice that of PCV13 policy implementation (Table 4). Active policy strategies clearly required higher budget investment (50–79 million USD per year for PCV13) than passive policy strategies. However, vaccine coverage was much higher in active policy strategies than in passive policy strategies [active policy (77%) vs. passive policy (25%)].

**Table 4:** Budget impact analysis of PCV13 stratified by policy types: (A) Passive policy (B) Active policy.

A. Passive policy						
Year	2022	2023	2024	2025	2026	Total
Total patients	8,185,581	696,159	725,731	793,299	833,138	11,233,908
Total eligible (exclude previous vaccination)	7,776,302	661,351	689,444	753,634	791,481	
Number of populations with acceptance	3,888,151	330,676	344,722	376,817	395,741	
Vaccine access rate (%)	0.20	0.30	0.40	0.50	0.60	
Number of accessible population with acceptance	777,630	99,203	137,889	188,409	237,444	1,440,575 (vaccine coverage of 13, 25% including previous vaccination)
*PCV13 full price (64.71 USD)*						
Total budget (USD)	50,318,235	6,419,121	8,922,397	12,191,378	15,364,346	93,215,478 Average per year (18,643,096)
*PCV13 reduced price (40.76 USD)*						
Total budget (USD)	31,698,616.01	4,043,807.67	5,620,778.37	7,680,114.70	9,678,966.54	58,722,283 Average per year (11,744,457)
*PCV13 reduced price (59.04 USD)*						
Total budget (USD)	45,909,059.60	5,856,640.78	8,140,565.15	11,123,098.98	14,018,033.21	85,047,398 Average per year (17,009,480)
*PPSV23 full price (29.44 USD)*						
Total budget (USD)	22,895,577	2,920,800	4,059,829	5,547,266	6,991,016	42,414,488.40 Average per year (8,482,898)

B. Active policy						
Year	2022	2023	2024	2025	2026	total
Total patients	8,185,581	7,694,831	6,939,307	6,067,172	5,155,998	11,233,908
Total eligible (exclude previous vaccination)	7,776,302	7,310,089	6,592,341	5,763,814	4,898,198	
Number of populations with acceptance	3,888,151	3,655,045	3,296,171	2,881,907	2,449,099	
Vaccine access rate (%)	0.20	0.30	0.40	0.50	0.60	
Number of accessible populations with acceptance	777,630	1,096,513	1,318,468	1,440,953	1,469,459	6,103,025 (vaccine coverage of 54, 77% including previous vaccination)
*PCV13 full price (64.71 USD)*						
Total budget (USD)	50,318,235	70,952,258	85,314,328	93,239,990	95,084,537	394,909,348 Average per year (78,981,870)
*PCV13 reduced price (40.76 USD)*						
Total budget (USD)	31,698,616.01	44,697,282.71	53,744,852.75	58,737,724.81	59,899,721.25	248,778,198 Average per year (49,755,640)
*PCV13 reduced price (59.04 USD)*						
Total budget (USD)	45,909,059.60	64,735,009.72	77,838,592.29	85,069,761.65	86,752,679.42	360,305,103 Average per year (72,061,021)
*PPSV23 full price (29.44 USD)*						
Total budget (USD)	22,895,577	32,284,378	38,819,343	42,425,642	43,264,939	179,689,879 Average per year (35,937,976)

## 4. Discussion

Cost-effectiveness analysis is a crucial information to support decision making for policy makers (45). This analysis has a critical role in low- and middle-income countries (LMICs) for prioritizing vaccines. Our analyses revealed that comparison one vaccine (PPSV23 or PCV13) over another showed that PCV13 are economical vaccine for all older adult Thai individuals when compared to PPSV23 and no vaccinations. These findings are consistent with previous analyses of the cost-effectiveness of these vaccines in adults aged ≥ 50 years residing in low- and middle-income countries (46). We anticipated that ICER between immunocompromised and non-immunocompromised subgroups were different. This is because input parameters between those are different, particularly vaccine efficacy and epidemiological data. Still, both are vaccine target group in clinical practice these day in Thailand. The main determinant factor was PCV13 efficacy on pneumococcal pneumonia across all populations according to the sensitivity analyses and CDC data which protection against pneumonia was lower than IPD. Consequently, when policy makers intend to conduct a similar analysis, they should carefully pay attention to input data of vaccine efficacy or effectiveness against pneumonia since they are the most sensitive parameter. Although the incremental cost among immunocompromised group was higher than healthy and chronic health conditions, the incremental QALY when PPSV23 compared to no vaccination were more than PCV13 compared to one another. This is because utility played a major sensitive factor in immunocompromised conditions according to our sensitivity analyses. Therefore, the direction of ICERs of scenario based on individual with immunocompromised conditions when comparing PPSV23 and no vaccination was lower; meanwhile, the direction of ICER PCV13 comparing one another was higher than that of healthy individuals or those with chronic health conditions.

The majority of previous studies indicated that PCV13 or PPSV23 were cost-saving in specific countries; however, our findings indicated that, using the current price of vaccines, PCV13 and PPSV23 were both cost-effective but not cost-saving. The prices of PCV13 must be reduced by 8.8–19.0% of actual price, depending on the age and conditions. The older adult has lower vaccination implementation costs than children (11). Moreover, the price of the vaccine is the primary barrier to pneumococcal vaccine implementation in middle-income countries (47, 48). Even though our study demonstrated that both vaccines are cost-effective, price reductions are still required to achieve cost-saving results. We anticipated that if we included the cost of complications from deteriorated medical condition resulting from pneumococcal infection, the ICER of vaccine would be lower.

The outcomes of studies comparing one vaccine (PCV13 or PPSV23) to another displayed PCV13 was cost-saving compared to PPSV23 (46). Our findings indicate that PCV13 is superior to PPSV23 because it extends life expectancy and is more effective. In addition, the probability of PCV13 being cost-saving was significantly greater than that of PPSV23. We proposed that PCV13 should be prioritized before PPSV23 in NIP. However, before vaccine implementation, stakeholders must engage in discussion. This is because BIA is typically preferred by the Thai government when the annual budget is less than 4.7 million USD (49–51). Based on our research, the Thai government must invest at least 8 million USD annually for PPSV23 and more than 12 million USD annually for PCV13. In addition, we designed active and passive policy alternatives to determine whether or not Thailand would reach its vaccine coverage goal. The Thai government must spend a substantial amount of money on active policy, but the high vaccine coverage rate and herd immunity threshold may be attained in a relatively short period. While the passive policy may hardly represent the real-world situations, its advantage is budget capability. We recommend that the costs of pneumococcal disease-burden reduction after vaccine and policy deployment be incorporated in our analysis when the Thai government considers this vaccination program. In addition, the duration of pneumococcal vaccination campaigns may be extended to reduce the annual fiscal burden.

Moreover, our results were sensitive to the vaccine efficacy against pneumonia among the Thai older adult. The efficacy of PCV13 against pneumonia was determined to be superior to that of PPSV23. Pneumococcal pneumonia is more prevalent than IPDs despite its restricted serotype coverage. In addition, PCV13 is significantly more effective against pneumonia than PPSV23. Our findings were consistent with those of Smith et al. (20).

Our research has several strengths. First, most of our input data were selected based on Thailand-specific information to produce data and make the outcomes more country-specific. The probabilistic sensitivity analytic model indicated that the constructed model was robust and valid. We also adopted the recent updated pneumococcal vaccine efficacy/effectiveness from the CDC model, which included several significant changes that were closed to real world data: (1) the duration of PCV protection was shorter and (2) vaccine efficacy/effectiveness was lower for PCV13 and PPSV23 in immunocompromised conditions. In addition, this model will be beneficial to the two-next generation pneumococcal vaccines implementation. Lastly, our BIAs incorporated vaccine uptake rate and were stratified by policy types, i.e., active or passive to create different scenarios for decision making. However, this study has some limitations. First, our model was developed using static model, whose results should be interpreted with caution. Second, herd immunity was not included in the model due to the low vaccine uptake rate. The model should be updated when the nationwide pneumococcal vaccine deployment among children reaches herd immunity threshold, or the valent vaccine are implemented. Lastly, as the absence of local data regarding the utility of adults with infection, the utility ratio of populations at high and average risk in the same age range were derived from population in Western countries.

In conclusion, PCV13 is cost-effective among Thai older adult. PCV13 should be prioritized first when both costs and benefits are considered. The cost of vaccines is the most significant barrier to national implementation in Thailand; therefore, negotiations with manufacturers is encouraged. A proactive policy can contribute rapidly to herd immunity in a community, but substantial resources are required. New generation of pneumococcal vaccines with a higher valency will soon be available in high-income countries. Further economic analysis of these new generation vaccines in the context of Thai society is needed.

## Data availability statement

The original contributions presented in the study are included in the article/supplementary material, further inquiries can be directed to the corresponding author.

## Author contributions

TN, CK, and PP contributed to the original idea and conceived the paper. TN wrote the initial draft of the paper under CK and PP guidance. SL, PR, VL, PC, JK, and KC contributed to the revision of the manuscript, and the final version was reviewed by TN, CK, and PP. The authors acknowledge CK’s essential intellectual contribution. All authors contributed to the article and approved the submitted version.

## Funding

This research was supported by Mahidol University (MU-miniRC grant: MU-MiniRC04/2564, fiscal year 2021; SL). The APC was funded by the Faculty of Tropical Medicine, Faculty of Medicine Siriraj Hospital Mahidol University, and Mahidol University.

## Conflict of interest

The authors declare that the research was conducted in the absence of any commercial or financial relationships that could be construed as a potential conflict of interest.

## Publisher’s note

All claims expressed in this article are solely those of the authors and do not necessarily represent those of their affiliated organizations, or those of the publisher, the editors and the reviewers. Any product that may be evaluated in this article, or claim that may be made by its manufacturer, is not guaranteed or endorsed by the publisher.
